# Community environment, cognitive impairment and dementia in later life: results from the Cognitive Function and Ageing Study

**DOI:** 10.1093/ageing/afv137

**Published:** 2015-10-13

**Authors:** Yu-Tzu Wu, A. Matthew Prina, Andrew P. Jones, Linda E. Barnes, Fiona E. Matthews, Carol Brayne

**Affiliations:** 1 Cambridge institute of Public Health, University of Cambridge, Cambridge, UK; 2 Centre for Global Mental Health, Health Services and Population Research, Institute of Psychiatry, King's College London, London, UK; 3 Norwich Medical School, University of East Anglia, Norwich, UK; 4 MRC Biostatistics Unit, University of Cambridge, Cambridge, UK

**Keywords:** cognitive impairment, dementia, neighbourhood/community environment, older people

## Abstract

**Background:** few studies have investigated the impact of the community environment, as distinct from area deprivation, on cognition in later life. This study explores cross-sectional associations between cognitive impairment and dementia and environmental features at the community level in older people.

**Method:** the postcodes of the 2,424 participants in the year-10 interview of the Cognitive Function and Ageing Study in England were mapped into small area level geographical units (Lower-layer Super Output Areas) and linked to environmental data in government statistics. Multilevel logistic regression was conducted to investigate associations between cognitive impairment (defined as MMSE ≤ 25), dementia (organicity level ≥3 in GMS-AGECAT) and community level measurements including area deprivation, natural environment, land use mix and crime. Sensitivity analyses tested the impact of people moving residence within the last two years.

**Results:** higher levels of area deprivation and crime were not significantly associated with cognitive impairment and dementia after accounting for individual level factors. Living in areas with high land use mix was significantly associated with a nearly 60% reduced odds of dementia (OR: 0.4; 95% CI: 0.2, 0.8) after adjusting for individual level factors and area deprivation, but there was no linear trend for cognitive impairment. Increased odds of dementia (OR: 2.2, 95% CI: 1.2, 4.2) and cognitive impairment (OR: 1.4, 95% CI: 1.0, 2.0) were found in the highest quartile of natural environment availability. Findings were robust to exclusion of the recently relocated.

**Conclusion:** features of land use have complex associations with cognitive impairment and dementia. Further investigations should focus on environmental influences on cognition to inform health and social policies.

## Introduction

With rapid increase in the number of older people, cognitive decline and dementia have become important health issues [1]. Longitudinal studies have investigated the epidemiology of dementia and cognitive impairment in community-based populations [2–4], identifying potential risk factors including lifestyle (physical activity, social interaction) and chronic conditions (vascular diseases, metabolic syndrome and depression) [5, 6]. These risk factors could however be moderated by the community environment acting as an additional determinant of health. Identifying environmental features related to cognition in later life may therefore reduce dementia occurrence by moderating individual risk factors.

**Figure 1.**
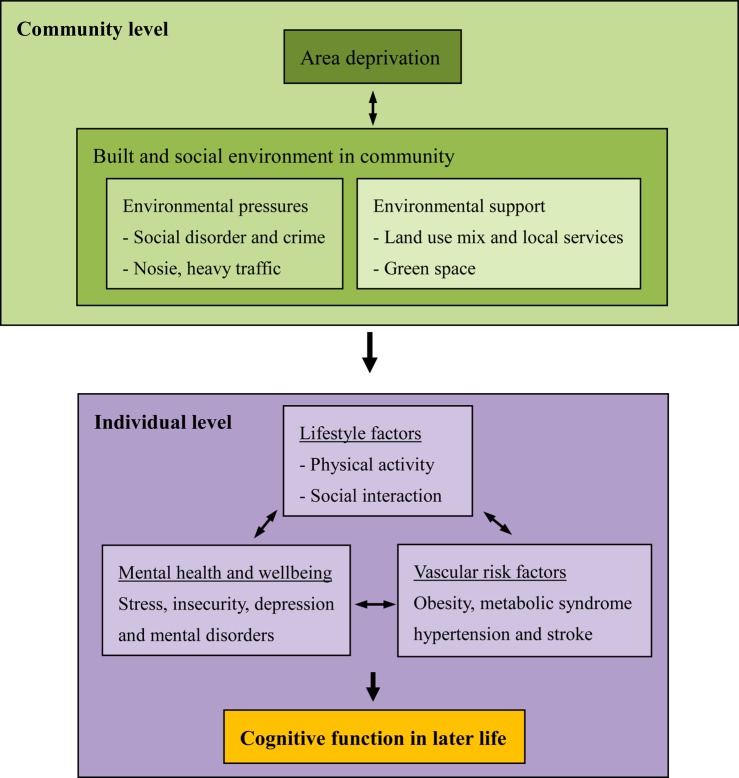
Theoretical framework of the pathway from community environment to cognitive function of older people.

A small number of studies have reported that older people living in more deprived areas have a higher risk of cognitive impairment or decline that persists after adjusting for individual demographic factors [7–9]. Since area deprivation is a proxy for built and social environmental features in communities, this highlights the potential influence of the community environment on cognitive function in later life, as described by the theoretical framework in Figure 1. A high level of area deprivation might be related to environmental pressures, such as crime, low greenspace availability and poor access to local services. Environmental factors could have a potential impact on individual lifestyles, with a consequent bearing on the risk of obesity and vascular diseases, as well as mental health and well-being [10–12]. For example, research has suggested that a high mix of land uses and availability of greenspace can encourage physical activity, which might reduce vascular risk factors for dementia as well as increase social interaction, providing cognitive stimulation for older adults [10, 11]. Alternatively, a high level of crime in local areas might have a negative impact on emotion and increase the risk of depression, a known risk factor for dementia [12]. Environmental features which support active ageing may therefore reduce risk of cognitive impairment and dementia while environmental pressures such as high crime rates might have the opposite effect.

This study builds on a previous review [9] and includes both compositional (area deprivation) and contextual measurements (features of land use and crime) to explore the role of built and social environmental features in cognition of older people. This is an early exploratory work, and hence the analysis focuses on the investigation of cross-sectional associations between community level factors, cognitive impairment and dementia using a large population-based study of older people in England.

## Method

### Study population

The Medical Research Council Cognitive Function and Ageing Study (CFAS) is a longitudinal population-based study investigating cognitive and physical decline of people aged 65 years and over in six centres across England and Wales (Liverpool, Cambridgeshire, Gwynedd, Newcastle upon Tyne, Nottingham and Oxford). Identical study design and measurement methods were used at each except Liverpool, which was excluded from this analysis. Full details of CFAS have been described elsewhere [13]. Briefly, community and institutionalised populations were sampled from General Practice Registers to capture equal sized samples of the age groups 65–74 and 75 years and over. Baseline in-home interviews were conducted between 1991 and 1994. Among 16,258 individuals invited for the study, 13,004 completed the initial screening interview with a response rate of 80%. The main follow-up waves included 1 year follow-up and a 2 year rescreen, new selection for assessment and further a 1 year follow-up, a 6 year follow-up of the assessed, an 8 year follow-up of a specific subgroup and a 10 year follow-up of the whole sample (see www.cfas.ac.uk). Due to limited environmental data in the 1990s, the analysis focuses on the 2,424 participants who attended the year-10 interview in 2001 from the four English centres (Cambridgeshire, Newcastle upon Tyne, Nottingham and Oxford). The Welsh centre (Gwynedd) was excluded due to the lack of comparable information on area deprivation.

### Individual level measurements

Socio-demographic information, including age, gender, education and social class was recorded at the baseline interview. Education was divided into two groups separating people with nine or fewer years of education and those with ten years and above. The longest occupation reported was used to classify the social class of each participant according to the Registrar General's occupation-based social class [14]. Participants with social class classifications I to IIINM were grouped as the ‘non-manual’ group while social class IIIM to V was grouped into the ‘manual’ group. The interview question ‘have you moved in the last two years?’ was used to identify recently relocated individuals.

Several chronic conditions which usually occurred in middle or later life are known to be related to cognitive impairment and dementia in older people [6]. The number of chronic illnesses, including vascular risk factors (hypertension, diabetes, stroke, heart attack, angina, low blood pressure) and sense impairment (hearing and vision impairment), were recorded based on self-reported information in the year-10 interview.

The interview included a structured assessment of cognitive function and mental status. Cognitive function was measured with the Mini-Mental State Examination (MMSE) [15]. Cognitive impairment here was defined as a MMSE score of 25 and below [16]. Dementia cases were defined as organicity level three and above using the Geriatric Mental Status and the algorithm of the Automatic Geriatric Examination for Computer Assisting Taxonomy [17].

### Community level measurements

Based on information from the National Statistics Postcode Directory, the postcodes of the year-10 participants were mapped to Lower-layer Super Output Areas (LSOA), a geographic unit developed for the collation of small area statistics following the 2001 UK Census, with an average of 1,500 residents [18]. In cases where postcodes from the year-10 interview were missing or incorrect, the full address was used to obtain complete postcodes from the Royal Mail, Google Maps and property websites.

Environmental data for each LSOA were obtained from published UK Government Neighbourhood Statistics (www.neighbourhood.statistics.gov.uk), a collection of small area level data across England. Area deprivation was measured by the English Index of Multiple Deprivation 2004 (IMD 2004), which was based on data collected in 2001 and 2002 [19]. The IMD summarised seven domains of characteristics related to deprivation including income, employment, education and training, health and disability, barriers to housing and services, the living environment and crime. The crime score, a summarised score of recorded crime data, was extracted from the crime domain of IMD. Measures of land use mix and the natural environment were derived for the residential area of each participant based on the Generalised Land Use 2001 dataset, which provided areas of different types of land use in all the LSOAs across England. The measure of land use mix was set to indicate the diversity of land use types in each LSOA. A high mix of land uses suggests the close integration of residential, commercial and recreational uses with a variety of facilities, services and resources in local areas. The calculation method followed that used in existing literature and employed a range from 0 (lowest heterogeneity of land use) to 1 (highest) [20]. The measure of the natural environment employed was the percentage of greenspace and private gardens in each LSOA.

### Analysis strategy

The association between community level measurements (area deprivation, land use mix, natural environment and crime), cognitive impairment and dementia was investigated by multilevel logistic regression taking individual level factors (age, gender, education, social class and the number of chronic illnesses) into account. To control for the potential influence of socioeconomic disadvantage and other correlated environmental factors, the association between features of land use (land use mix and natural environment), cognitive impairment and dementia was further adjusted for area deprivation. Since those who had recently relocated would have less exposure to local environmental characteristics, a sensitivity analysis was carried out by excluding the recent relocated.

## Results

The minimum age of the 2,424 participants was 74 years with a mean age of 81.7 (standard deviation 5.1) (Table 1). The crude prevalence of cognitive impairment (MMSE ≤ 25) and dementia in this population were 33.7 and 7.6% respectively. Older age, being female and lower education and social class were associated with a higher prevalence of cognitive impairment and dementia.

**Table 1.** Descriptive statistics of the study population

Category	Cognitive impairment (MMSE ≤ 25)	Dementia	Total
*N*	809 (33.4)	185 (7.6)	2,424
Missing	25 (1.0)	3 (0.1)	
Age			
74–79	210 (21.2)	29 (2.9)	992
80–84	257 (33.1)	44 (5.6)	776
85–89	215 (49.0)	63 (14.4)	439
90+	127 (58.5)	49 (22.6)	217
Gender			
Men	248 (26.0)	53 (5.6)	953
Women	561 (38.1)	132 (9.0)	1,471
Education			
>9 years	241 (25.0)	43 (4.5)	966
≤9 years	565 (38.9)	141 (9.7)	1,452
Social class			
Non-manual	291 (26.2)	66 (5.9)	1,111
Manual	510 (39.4)	118 (9.2)	1,295
Number of chronic illnesses			
None	236 (34.9)	106 (15.7)	676
One	254 (31.8)	36 (4.5)	799
Two and more	319 (33.6)	43 (4.5)	949

### The association between the community environment and cognition in later life

Although higher odds of cognitive impairment and dementia were found in the most deprived areas, the association was less clear after controlling for individual level factors (Model 2, Table 2). The associations between land use mix, natural environment and cognitive impairment were generally not linear. For land use mix, the odds of cognitive impairment decreased from the first to the third quartile [odds ratio (OR) in the third quartile: 0.69, 95%CI: 0.51, 0.95] but then slightly increased in the fourth quartile (OR: 0.86, 95%CI: 0.63, 1.16), the highest level of land use mix. A nearly 40% lower odds of dementia was found in the second to fourth quartile of land use mix but the association was not statistically significant. For the natural environment, there was a higher odds ratio of cognitive impairment and dementia in the fourth quartile compared with the first, although none of odds ratios were significantly different from the reference category. The association between crime, cognitive impairment and dementia was unclear after taking individual level factors into account. Excluding those who had moved residence in the past two years did not substantially influence estimates.

**Table 2.** The associations between cognitive impairment and dementia, area deprivation, built and social environmental features

		Cognitive impairment (MMSE ≤ 25)			Dementia		
		Model 1 OR (95% CI)	Model 2 OR (95% CI)	Model 3 OR (95% CI)	Model 1 OR (95% CI)	Model 2 OR (95% CI)	Model 3 OR (95% CI)
Area deprivation							
(Least deprived)	Q1 (ref.)	1.00	1.00		1.00	1.00	
	Q2	1.38 (1.02, 1.86)	1.21 (0.87, 1.68)		1.19 (0.65, 2.17)	1.05 (0.55, 2.00)	
	Q3	1.27 (0.94, 1.72)	1.03 (0.74, 1.42)		1.42 (0.79, 2.54)	1.19 (0.64, 2.22)	
(Most deprived)	Q4	1.50 (1.12, 2.00)	1.16 (0.84, 1.61)		1.58 (0.91, 2.74)	1.39 (0.76, 2.56)	
			*P* = 0.63			*P* = 0.23	
Built environment							
Land use mix							
(Lowest)	Q1 (ref.)	1.00	1.00	1.00	1.00	1.00	1.00
	Q2	0.81 (0.60, 1.09)	0.76 (0.55, 1.04)	0.75 (0.55, 1.03)	0.63 (0.35, 1.12)	0.60 (0.33, 1.09)	0.54 (0.29, 0.98)
	Q3	0.72 (0.53, 0.98)	0.69 (0.51, 0.95)	0.66 (0.48, 0.92)	0.65 (0.36, 1.15)	0.68 (0.37, 1.23)	0.57 (0.31, 1.04)
(Highest)	Q4	0.92 (0.69, 1.22)	0.86 (0.63, 1.16)	0.81 (0.59, 1.12)	0.59 (0.34, 1.04)	0.58 (0.32, 1.03)	0.44 (0.23, 0.82)
			*P* = 0.39	*P* = 0.24		*P* = 0.11	*P* = 0.02
Natural environment							
(Lowest)	Q1 (ref.)	1.00	1.00	1.00	1.00	1.00	1.00
	Q2	0.76 (0.57, 1.02)	0.78 (0.57, 1.04)	0.80 (0.59, 1.08)	0.80 (0.47, 1.37)	0.96 (0.54, 1.71)	1.05 (0.60, 1.86)
	Q3	0.86 (0.65, 1.14)	0.99 (0.73, 1.33)	1.04 (0.77, 1.42)	0.76 (0.44, 1.31)	0.95 (0.53, 1.70)	1.16 (0.64, 2.10)
(Highest)	Q4	1.12 (0.83, 1.51)	1.28 (0.93, 1.75)	1.41 (1.00, 1.98)	1.38 (0.78, 2.42)	1.64 (0.91, 2.97)	2.23 (1.17, 4.24)
			*P* = 0.08	*P* = 0.03^a^		*P* = 0.15	*P* = 0.02
Social environment							
Crime (Least)	Q1 (ref.)	1.00	1.00		1.00	1.00	
	Q2	1.06 (0.78, 1.45)	0.94 (0.68, 1.30)		1.65 (0.90, 3.02)	1.34 (0.71, 2.54)	
	Q3	1.20 (0.89, 1.63)	0.97 (0.71, 1.34)		1.91 (1.05, 3.48)	1.55 (0.83, 2.89)	
(Most)	Q4	1.23 (0.91, 1.64)	0.96 (0.70, 1.31)		1.52 (0.85, 2.73)	1.15 (0.62, 2.12)	
			*P* = 0.88			*P* = 0.70	

Model 1: Unadjusted estimates of odds ratio (OR) of individual and community level factors.

Model 2: The estimates of OR were adjusted for individual level factors (age, gender, education, social class and number of chronic illnesses).

Model 3: The estimates of OR were further adjusted for individual level factors and area deprivation.

*P*: *P*-value of test for trend.

^a^Although both test for trend (*P* = 0.03) and heterogeneity (*P* = 0.01) were significant, the *P*-value of likelihood ratio test for linearity was 0.04, which indicated that the relationship was more likely to be non-linear. The trend might be driven by the higher odds in the fourth quartile.

After further adjusting for area deprivation, the odds of dementia significantly decreased with higher levels of land use mix (Model 3, Table 2). Living in the highest quartile of land use mix was associated with a 60% lower odds of dementia (OR: 0.44, 95%CI: 0.23, 0.82). There was no such trend for cognitive impairment. A higher odds of dementia (OR: 2.23, 95%CI: 1.17, 4.23) and cognitive impairment (OR: 1.41, 95%CI: 1.00, 1.98) was found in the highest quartile of natural environment availability with a significant test for trend.

## Discussion

### Main findings

This study explored the potential impact of the community environment on cognitive impairment and dementia in later life, investigating associations with built and social environmental features in a diverse sample of communities across England. No significant associations between area deprivation, crime, cognitive impairment and dementia were found in this population aged 74 and over. Living in areas in the highest quartile of land use mix was however significantly associated with a nearly 60% reduced odds of dementia after adjusting for individual level factors and area deprivation. Higher odds of dementia and cognitive impairment were found in the highest quartile of natural environment availability. The associations between land use mix, natural environment and cognitive impairment did not appear to be linear.

### Strengths and limitations

Compared with previous studies [7, 9], this study further included contextual measurements from independent data sources to identify important environmental features related to cognitive impairment and dementia in later life. The multicentre study design of CFAS included older people living in diverse community environments across England and a structured psychiatric interview was used to maintain consistency of diagnostic standards.

As with other cross-sectional studies, the causal directions could not be examined, and the direction of association may be reversed if people with cognitive impairment or dementia moved to communities with supportive environmental features. The lack of environmental data for the CFAS baseline in 1991 limited our ability to investigate longitudinal associations.

This study population included nearly 2,500 older people but small numbers of dementia cases still limited our ability to detect significant differences across different types of community environments. The population studied here were survivors and respondents from the baseline interview ten years earlier. Previous CFAS analyses on longitudinal attrition reveal higher refusal rate in those with poor cognitive ability and low education and increased likelihood of relocation in those living deprived areas and rural settings [21, 22]. Although the percentage of refused or moved populations was relatively low in CFAS interviews (less than 20%), those with disadvantaged socioeconomic status, poor cognition and health status more were likely to drop out or die over the 10 years, therefore this analysis might have selection bias. People with dementia do move to institutions and could have different interactions with community environments. Although this study did not identify the institutionalised population, only about 3% of the sample reported moving to institutions in the previous two years [23]. The impact of these moves on the findings is therefore likely to be small.

Higher number of chronic illnesses was associated with lower odds of cognitive impairment and dementia. This may be driven by reporting bias whereby people with dementia might have difficulties reporting their full medical history. The influence of co-morbidity on the associations presented might therefore not be completely controlled for but the adjustment of different types of chronic conditions did not considerably change the results (Table S1, Appendix). Lifestyle factors were not recorded in the year-10 interview. As factors such as physical activity have been associated with neighbourhood environments, there may be unconditional confounding associated with their omission.

### The community environment and cognition in later life

Although previous studies suggest a positive relationship between area deprivation and cognitive impairment, this analysis did not replicate those findings in this older population [7]. This might indicate that the influence of area deprivation can be, to a certain extent, attributed to individual socioeconomic factors. Since compositional measurements such as deprivation scores are typically strongly correlated with individual socioeconomic status, it is difficult to disentangle effects of place from individual level factors [24].

A high level of land use mix was associated with decreased odds of dementia. Older people living in areas with mixed land use might have better access to local services, potentially increasing social interactions and cognitive stimulation. However, the odds of cognitive impairment actually slightly increased in the highest quartile of land use mix after adjusting for area deprivation. It may be that communities with high land use mix support people with cognitive impairment to remain living in local areas whilst those with dementia are more likely to move away from such environments.

A higher availability of greenspace in local areas was associated with higher odds of dementia and cognitive impairment. This finding may be spurious although, alternatively, it might suggest that living in communities with extremely high natural environment availability could be related to isolation, barriers to accessing local services and a consequent lack of cognitive stimulation. Another possibility is that high natural environment availability supports people with cognitive impairment and dementia to remain in their communities.

Evidence in the literature has reported that fear of crime and insecurity may limit the mobility of older people and increase the risk of depression [25, 26]. However, the association between crime, cognitive impairment and dementia was unclear in this study. Perceptions of crime and insecurity are likely to vary between individuals and there is equivocal evidence in the criminology literature about whether older people experience more fear of crime compared with younger age groups [27, 28].

### Future research directions

In addition to individual risk factors, this study found some evidence to suggest the community environment may influence cognition in later life. A greater focus on addressing environmental influences could help efforts to reduce the risk of cognitive impairment and dementia in older people.

Cognitive decline is a continuous and dynamic condition. The interaction with community environments in later life may change with increased age and functional decline. Studies employing global positioning systems, which track mobility patterns of individuals in the environment, are becoming widespread to better understand environmental influences on physical activity and the technologies also offer much potential in this field [29]. Potential mechanisms need to be further explored in longitudinal studies with complete information on residential relocation, lifestyle, plus physical and mental health status over time. Future studies could also include more detailed information on environmental features, such as pavement conditions and public transport availability, both of which might influence outdoor mobility and active ageing [30].

**Key points**

- Area deprivation and crime were not significantly associated with cognitive impairment and dementia.
- The associations between land use mix, natural environment availability and cognitive impairment did not appear to be linear.
- Unfavourable environmental features might limit the daily activities of older people increasing the risk of cognitive decline.

## Supplementary Material

Supplementary Data
