# Supplementary material for: Community environment, cognitive impairment and dementia in later life: results from the Cognitive Function and Ageing Study
Source: Age Ageing. 2015 Oct 13;44(6):1005–11. doi: 10.1093/ageing/afv137 (PMC4621236; doi:10.1093/ageing/afv137)
Supplement: Supplementary Data [file supp_44_6_1005__index.html]

Community environment, cognitive impairment and dementia in later life: results from the Cognitive Function and Ageing Study — Supplementary Data 

# Community environment, cognitive impairment and dementia in later life: results from the Cognitive Function and Ageing Study

## Supplementary Data

Supplementary Data

- Supplementary Data - Pdf file
