# Supplementary material for: Community environment, cognitive impairment and dementia in later life: results from the Cognitive Function and Ageing Study
Source: Age Ageing. 2015 Oct 13;44(6):1005–11. doi: 10.1093/ageing/afv137 (PMC4621236; doi:10.1093/ageing/afv137)
Supplement: Supplementary Data [file supp_afv137_afv137supp.pdf]

Community environment, cognitive impairment and dementia in later life: results from the Cognitive Function and Ageing Study

Appendix S1: The adjustment for chronic conditions

To adjust for potential influence of chronic conditions, a sensitivity analysis of including different measures of health status was conducted. The results of Model 2 were adjusted for socio-demographic factors and number of co-morbidity. Instead of using the count variable for co-morbidity, Model 2a included chronic conditions individually (hypertension, diabetes, stroke, heart attack, angina, low blood pressure, hearing/visual impairment and depression) for further adjustment. To address the issue of self-reported information on chronic illnesses, Model 2b included functional impairment (ADL/IADL), which is a strong indicator of decline in physical health caused by various chronic conditions. Although functional impairment might be an outcome of cognitive impairment or dementia, it could be a robust measure of poor health status.

The results of these three models are reported in **Table S1**. The direction and strength of association between community level factors, cognitive impairment and dementia did not change considerably using different types of adjustment for chronic conditions.

Table S1 The association between community level factors, cognitive impairment and dementia adjusting for socio-demographic factors and chronic conditions

		Cognitive impairment (MMSE≤25)			Dementia		
		Model 2	Model 2a	Model 2b	Model 2	Model 2a	Model 2b
		OR (95% CI)	OR (95% CI)	OR (95% CI)	OR (95% CI)	OR (95% CI)	OR (95% CI)
**Area deprivation**							
(Least deprived)	Q1 (ref.)	1.00	1.00	1.00	1.00	1.00	1.00
	Q2	1.21 (0.87, 1.68)	1.19 (0.85, 1.66)	1.13 (0.80, 1.59)	1.05 (0.55, 2.00)	0.97 (0.51, 1.86)	0.83 (0.44, 1.56)
	Q3	1.03 (0.74, 1.42)	0.99 (0.71, 1.38)	0.91 (0.65, 1.28)	1.19 (0.64, 2.22)	1.05 (0.56, 1.96)	0.86 (0.46, 1.59)
(Most deprived)	Q4	1.16 (0.84, 1.61)	1.11 (0.80, 1.54)	0.95 (0.68, 1.34)	1.39 (0.76, 2.56)	1.16 (0.63, 2.14)	1.06 (0.58, 1.94)
p.		0.63	0.87	0.47	0.23	0.57	0.71
**Built environment**							
Land use mix (Lowest)	Q1 (ref.)	1.00	1.00	1.00	1.00	1.00	1.00
	Q2	0.76 (0.55, 1.04)	0.74 (0.54, 1.02)	0.74 (0.53, 1.02)	0.60 (0.33, 1.09)	0.60 (0.33, 1.10)	0.71 (0.40, 1.26)
	Q3	0.69 (0.51, 0.95)	0.68 (0.49, 0.94)	0.65 (0.47, 0.90)	0.68 (0.37, 1.23)	0.60 (0.33, 1.10)	0.62 (0.35, 1.10)
(Highest)	Q4	0.86 (0.63, 1.16)	0.86 (0.64, 1.16)	0.81 (0.59, 1.10)	0.58 (0.32, 1.03)	0.56 (0.31, 1.00)	0.57 (0.33, 0.99)
p.		0.39	0.40	0.21	0.11	0.07	0.04
Natural environment (Lowest)	Q1 (ref.)	1.00	1.00	1.00	1.00	1.00	1.00
	Q2	0.78 (0.57, 1.04)	0.77 (0.57, 1.04)	0.80 (0.59, 1.10)	0.96 (0.54, 1.71)	0.92 (0.52, 1.64)	0.95 (0.54, 1.66)
	Q3	0.99 (0.73, 1.33)	1.00 (0.74, 1.34)	1.04 (0.76, 1.41)	0.95 (0.53, 1.70)	0.96 (0.54, 1.72)	1.06 (0.60, 1.88)
(Highest)	Q4	1.28 (0.93, 1.75)	1.31 (0.96, 1.80)	1.42 (1.03, 1.96)	1.64 (0.91, 2.97)	1.75 (0.97, 3.17)	1.77 (1.02, 3.08)
p.		0.08	0.06	0.02	0.15	0.10	0.05
**Social environment**							
Crime (Least)	Q1 (ref.)	1.00	1.00	1.00	1.00	1.00	1.00
	Q2	0.94 (0.68, 1.30)	0.93 (0.67, 1.29)	0.92 (0.66, 1.28)	1.34 (0.71, 2.54)	1.36 (0.72, 2.58)	1.47 (0.78, 2.79)
	Q3	0.97 (0.71, 1.34)	0.94 (0.68, 1.30)	0.94 (0.67, 1.30)	1.55 (0.83, 2.89)	1.48 (0.79, 2.79)	1.67 (0.88, 3.18)
(Most)	Q4	0.96 (0.70, 1.31)	0.94 (0.69, 1.29)	0.89 (0.64, 1.23)	1.15 (0.62, 2.12)	1.04 (0.56, 1.94)	1.25 (0.67, 2.33)
p.		0.88	0.77	0.51	0.70	0.99	0.57

Model 2: adjusted for age, gender, education, social class and number of chronic illnesses

Model 2a: adjusted for age, gender, education, social class, hypertension, diabetes, stroke, heart attack, angina, low blood pressure, hearing/visual impairment and depression

Model 2b: adjusted for age, gender, education, social class and functional impairment (ADL/IADL)

p.: p-value of test for trend
